# Comparison of an Offline SPE–GC–MS and Online HS–SPME–GC–MS Method for the Analysis of Volatile Terpenoids in Wine

**DOI:** 10.3390/molecules25030657

**Published:** 2020-02-04

**Authors:** Cody Williams, Astrid Buica

**Affiliations:** Department of Viticulture and Oenology, Stellenbosch University, Stellenbosch 7600, South Africa; codyw@sun.ac.za

**Keywords:** wine, terpenoid, terpene, solid-phase extraction, solid-phase microextraction, gas chromatography, mass spectrometry

## Abstract

The aroma profile is an important marker for wine quality. Various classes of compounds are responsible for the aroma of wine, and one such class is terpenoids. In the context of this work, a validated gas chromatography–mass spectrometry (GC–MS) method for the quantitation of terpenoids in red and white wine using headspace solid-phase microextraction (HS–SPME) and solid-phase extraction (SPE) was established. Calibrations were performed in the respective base wine using both sample preparation methods. The linearity, precision and accuracy evaluated for the respective matrices were excellent for both sample preparations. However, the HS–SPME approach was more sensitive and more accurate. For both sample preparations, the quantification limits were lower than the odor thresholds in wine. The terpenoid concentrations (µg/L) were evaluated for 13 white wines using both sample preparation methods. Importantly, the online HS–SPME approach was more sensitive than the offline SPE method. The major terpenoids identified in the white wines evaluated were linalool (0.2–63 µg/L), geraniol (nd–66 µg/L) and α-terpineol (nd–85 µg/L).

## 1. Introduction

Aroma is an important parameter when evaluating the quality of grapes and wine [1]. The aroma profile is attributed to a variety of compounds which can be separated into various classes based on their chemical structure [2]. Selected classes include esters (‘fruity’/’floral’) [3], fatty acids (‘rancid’) [4,5], C_6_ alcohols and aldehydes (‘leafy’, ‘green’) [6], methoxypyrazines (‘green capsicum’) [7], varietal thiols (‘grapefruit’, ‘guava’, ‘granadilla’) [8], volatile phenols (‘smoke’) [9], reductive sulfur compounds (‘cabbage’) [10], lactones (‘wood’) [11], carbonyl compounds (‘creamy’) [12], and terpenoids (‘floral’) [2,13]. In the context of this work, terpenoids and C_13_ norisoprenoids are of particular importance, as they contribute to some highly desirable descriptors such as ‘floral’, ‘raspberry’, ‘tobacco’, ‘honey’, and ‘citrus’ notes [14]. 

Terpenes are by definition isoprenoids, which consist of the basic C_5_H_8_ unit, bound in a head-to-tail manner. The most prominent forms in the aroma profile of wine are the C_10_-monoterpenes, C_15_-sesquiterpenes and C_13_-norisoprenoids [15,16]. Terpenoids are secondary plant constituents which are mainly formed via a biosynthenthetic pathway; on the other hand, C_13_-norisoprenoids are usually derived from the oxidative degradation of diterpenes and carotenoids [17,18]. Although the presence of these aroma compounds is highly sought after in wines, the complexity of the wine matrix accompanied by the inherent low concentrations in wine already indicates the difficulty in the analysis of these compounds. The isolation of terpenoids often includes a pre-concentration step and/or the use of highly sensitive instrumentation [19]. Due to the possibility of terpene oxidation and rearrangements, the analysis technique should be rapid, simple and robust [2,20,21,22]. Terpenoid identification in wines and grapes has been discussed in reviews [23,24,25] and the various sample preparation strategies such as liquid–liquid extraction (LLE), liquid–liquid microextraction (LLME), solid-phase extraction (SPE), headspace-solid-phase microextraction (HS–SPME), stirrer bar sorptive extraction (SBSE) and purge-and-trap methods are well documented [1,14,19,22,26,27,28,29,30,31].

Although LLE and SPE are highly versatile and easily accessible techniques, common drawbacks include the generation of organic solvent waste, the use of large volumes of wine often required for sufficient concentration, and the fact that the sample preparation methods are generally offline, which in turn results in longer sample preparation time. In contrast, online HS–SPME is a technique developed in the 90s, which makes use of a fused-silica fiber often coated with a suitable stationary phase [32]. In comparison to the above-mentioned techniques, HS–SPME is generally faster and more efficient, more sensitive and is solvent-free [32]. 

**Scheme 1:**
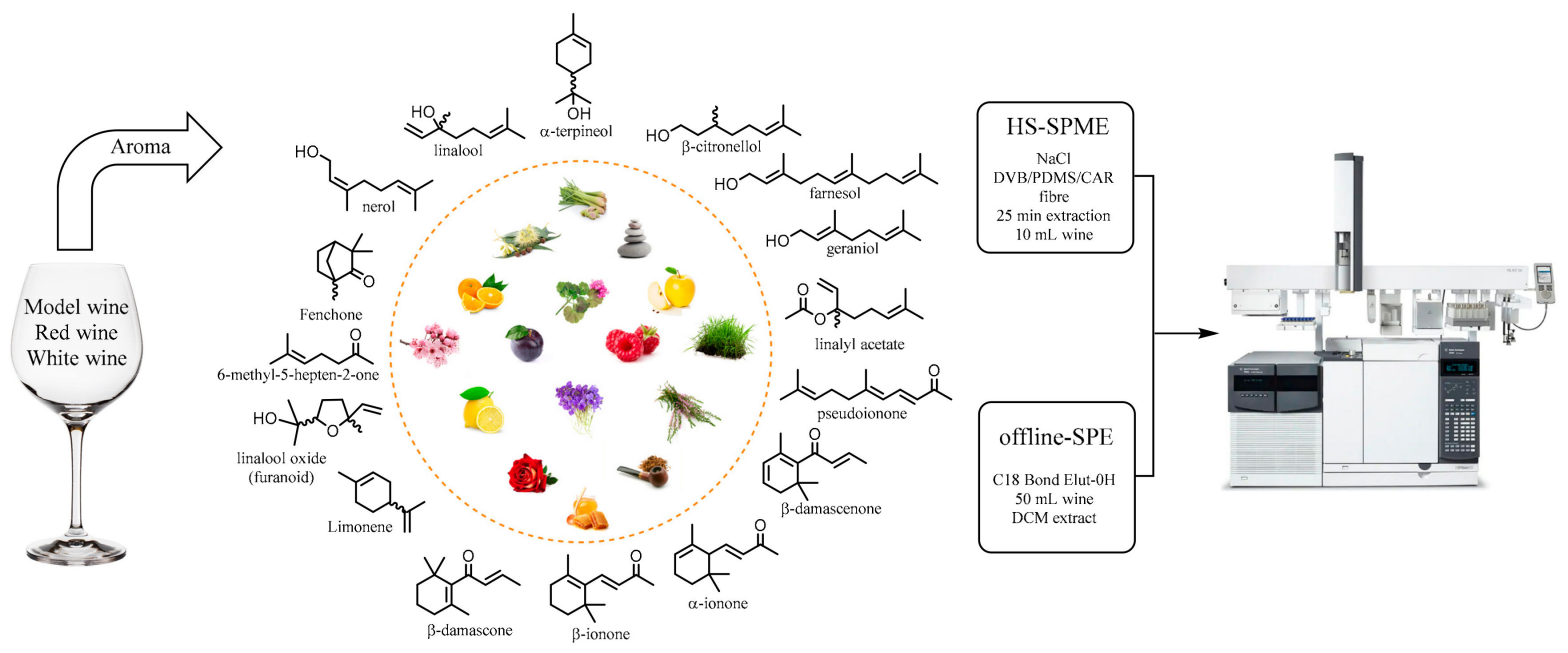
Schematic for the online headspace – solid-phase microextraction (HS–SPME) and offline solid phase extraction –gas chromatography – mass spectrometry (SPE–GC–MS) analyses of free terpenoids in wine.

As mentioned before, the aroma profile of a wine consists of a myriad of volatile compounds and the common technique for their analysis is gas chromatography with either flame-ionization (GC–FID) or mass spectrometry detection (GC–MS). Due to the complexity of wine, mass spectrometry is commonly used as an additional source of analyte confirmation to the more general retention time. As methods for the quantitation of terpenoids have been reported using both SPE and HS–SPME, there is a lack of literature on the comparison of the performance of these sample preparations using SPE vs. HS–SPME. Herein, we report the development and method performance parameters of an offline SPE–GC–MS and online HS–SPME–GC–MS method for the analysis of free terpenoids in red, white and model wines and application of both sample preparation methods in the analysis of free terpenoids in white wine (Scheme 1). 

## 2. Results and Discussion

### 2.1. Sample Preparation

The sample preparation used in this method for wine was rapid, simple, and resulted in improved sensitivity when compared to literature. Different methods proposed in literature use large sample volumes (up to 100 mL) [2,33], large volumes of organic solvent (up to 40 mL) [33,34], long extraction times (up to 60 min) [17] and long analysis times (up to 118 min) [17,33,35,36,37,38]. In comparison to the sample preparation methods in the current study, the volume of wines for the offline SPE sample preparation is 50 mL and the volume of organic solvent used is 10 mL. The online HS–SPME method uses 10 mL of wine and the samples are extracted for 25 min. The instrument run time for both sample preparation methods is 49 min (Table 1). 

### 2.2. Comparison of the Figures of Merit for Method Testing Parameters 

The sample preparation methods were compared with respect to the qualitative (selectivity) and quantitative (linearity, precision, and accuracy) parameters.

The selectivity of the sample preparation methods was evaluated by comparing the terpenoids peaks in the chromatogram, in the presence and absence of interferences from the matrix. Retention indices were calculated for each compound and used for comparison purposes. The offline SPE (Figure S1a) and online HS–SPME (Figure S1b) total ion chromatograms in spiked model, spiked dearomatized white and a real white wine are provided in the Supplementary Materials (Figure S1). When comparing the sample preparations, notably more chemical interferences were observed using the HS–SPME approach. The rationale for this is that the DVB/PDMS/CAR fiber is commonly used for untargeted analyses due to its good extraction capability of a wide range of analytes (lower selectivity), which extrapolates to the observation of more chemical noise in the chromatography [54]. Regardless of the interferences observed, the compounds could still be quantified. A total of 20 compounds was quantified when using the method in the current study.

Both sample preparation methods showed excellent linear response in all of the matrices tested, as measured by the correlation coefficients (R^2^). In the context of calibration curves, the slope in all of the matrices were similar, which implies that a calibration may be performed in model wine and subsequently unknowns may be quantified with minor over- or underestimations in wines. For a practical determination of terpenoids, the analytical method should be able to measure concentrations below the odor threshold, which would allow for combined sensory and chemistry experiments. Notably, the LOQ for all terpenoids was below the ODTs in wine (Table 1). In addition, the LODs for both sample preparation methods were lower than those reported in literature. The limit of detection (LOD) and limit of quantitation (LOQ) for both sample preparations in the respective matrices are reported in Table 2. Notably, the LOD for the online HS–SPME method was much lower in comparison to the offline SPE method (as reported back to the original 50 mL sample) for most compounds, excluding 6-methyl-5-hepten-2-ol and geraniol, in model and white wine. Conversely, the LOD in red wines was higher for HS–SPME in comparison to SPE; this could be explained by the presence of more noise observed in the red wine samples, resulting in lower signal to noise ratios (SNRs) and consequently higher calculated LOD’s. 

The precision for the entire method (sample preparation and instrumental method) was evaluated by repeating the extraction of terpenoids at two calibration levels. The repeatability (expressed as RSD) was calculated based on concentrations and retention times of the analytes (Table 2). For both sample preparation methods, the retention time RSD was less than 0.6%. The calculated concentration repeatability was generally better for the online HS–SPME method in comparison to the off-line SPE method which is explained by the inherent operator influence for the entire offline SPE procedure. With respect to the matrices tested, the RSD was on average higher in model wine in comparison to white and red wine for both SPE and HS–SPME. The combined sample preparation and instrumental precision were acceptable for the determination of terpenoids in wine (RSD < 9% for SPE and <17% for HS–SPME). 

Accuracy of the HS–SPME and offline SPE sample preparation methods was determined via recovery tests, which allow for the quantitation of the analytes in the absence and presence of interferences. The recovery values in all matrices for both methods ranged between 81 and 119 % using 3-octanol as the internal standard. The recovery in both white and red wines were acceptable for the SPE method as compared to Piñeiro et al. [19] and Dziadas and Jelen [2]. For the HS–SPME method, the recovery of selected terpenoids in Madeira wine reported by Marques and co-workers [17,28] ranged from 54 to 115%.The current study indicates that the recovery in white wine was generally better for nerol, β-damascenone, geraniol, α-ionone and β-ionone when compared to work by Marques and co-workers [17,28].

Practicaly, HS–SPME requires a headspace autosampler, which is expensive tool and it is not present in all analytical laboratories. Hence, based on the method performance parameters examined, the offline SPE method was considered as a practical alternative tool in the analysis of terpenes in wine which resulted in acceptable method performance parameters.

### 2.3. Internal Standard Comparison

It is considered ideal to use deuterated compounds as internal standards to model the behavior of the analytes of interest throughout the analytical method when MS is used as a detector. Due to the high cost and rare nature of deuterated terpenoid standards, the work aimed to examine the differences between 3-octanol and 2,6-dimethyl-6-hepten-2-ol as practical and commercially available internal standards. Initially 2-octanol was used due to its precedence in literature [14,19,37,43,54]; however, co-elution was observed with fenchone, which prompted the use 3-octanol as an internal standard instead [28,55]. 2,6-Dimethyl-6-hepten-2-ol was also selected based on its chemical structure, as it contains an isoprene unit, which is inherent to terpenoid compounds. The method performance data showed that 3-octanol was well adapted to both the SPE and HS–SPME approaches for the analysis of terpenoids in wine, which suggested that the use of deuterated analyte analogues was not essential in this case. The combined sample preparation and instrument repeatability values were better using 2,6-dimethyl-6-hepten-2-ol (Table S1, see Supplementary Materials). This observation was expected as 2,6-dimethyl-6-hepten-2-ol is structurally more similar to the terpenoids than 3-octanol, and hence the former would account for any analyte losses during the sample preparation and instrument analyses. A similar trend was observed when comparing the recoveries for the different internal standards, 2,6-dimethyl-6-hepten-2-ol internal standard modelled the extraction process better than 3-octanol. This was observed for most compounds except for the linalool oxides and linalyl acetate when compared using both sample preparation methods. Although 2,6-dimethyl-6-hepten-2-ol internal standard outperformed 3-octanol, the former is no longer commercially available. Furthermore, the method performance using 3-octanol was still acceptable. Therefore, due to its availability, precedence in literature, and means to compare it to future work, 3-octanol was chosen as the internal standard for the further analyses of terpenoids in wine samples. 

### 2.4. Volatile Terpenoid Quantification in White Wine

The procedures developed were successfully applied in the determination of volatile terpenoids in white wine and consequently compared. The concentrations determined by SPE and HS–SPME are shown in Table 3. Using the SPE method, the major terpenoids present were linalool (range 0.6–64 µg/L), α -terpineol (range nd–86 µg/L), geraniol (range nd–66 µg/L) and cis-linalool oxide (range 0.07–31 µg/L). The total terpenoid concentrations for the SPE sample preparation method ranged from 13 to 232 µg/L. Using the HS–SPME sample preparation method, the total terpenoid concentrations ranged from 15 to 192 µg/L. The concentration ranges are comparable to terpenoid concentration found in non-aromatic white wines [2,13,17,19,56]. The major constituents in the wines when using the HS–SPME method were linalool (range 0.09-38 µg/L), α-terpineol (range 0.9–56 µg/L), geraniol (range 3.2–79 µg/L) and cis-linalool oxide (range 2.3–21 µg/L). In comparison to the SPE method, ß-ionone was quantified in 11 more wines and trans-pseudoionone was also quantified in 12 more wines when using the HS–SPME method. These observations are in line with the method performance data, which reiterates that the HS–SPME method is more sensitive than the SPE method. There are various reports on the quantitation of pseudoionone as a sum of the steroisomers in different matrices [18,40]. To the best of our knowledge, herein is the first report of the quantitation of individual cis/trans pseudoionone isomers in wine. The trans-pseudoionone concentrations ranged from nd to 1.1 µg/L and cis-pseudoionone concentrations ranged from nd to 1.48 µg/L. 

## 3. Materials and Methods 

### 3.1. Chemicals 

Sodium chloride (99.5%), tartaric acid (99.5%), ethanol CHROMASOLV® (99.8%), dichloromethane (DCM) (99.8%), methanol (99.9%), sodium sulfate (99%), limonene (97%), 6-methyl-5-hepten-2-one (99%), fenchone (98%), linalool oxide (mixture of *cis* and *trans* furanoid isomers), linalool (97%), α-terpineol (98.5%), nerol (97%), geraniol (98%), β-damascone (90%), β-damascenone (98%), α-ionone (90%), β-ionone (96%), pseudoionone (mixture of *cis* and *trans* isomers, 97%), farnesol (mixture of isomers, 95%), 3-octanol (99%) and 2,6-dimethyl-6-hepten-2-ol (96%) were purchased from Sigma-Aldrich (St. Louis, MO, USA). Linalyl acetate (95%) and β-citronellol (mixture of enantiomers, 99%) were purchased from Fluka Chemika (Buchs, Switzerland).

HF-Bond Elut LRC C_18_-OH SPE cartridges (500 mg) were purchased from Agilent technologies (Santa Clara, CA, USA) and the 50/30 µm DVB/CAR/PDMS stableflex SPME fiber assembly (23 Ga) was purchased from Supelco® (Bellefonte, PA, USA). UPLC water was obtained from a Milli-Q filtration system (Millipore Filter Cor., Bedford, MA, USA). Model wine contained 12% (*v*/*v*) ethanol and 5 g/L tartaric acid, to which the pH was adjusted to 3.5 with sodium hydroxide (Sigma-Aldrich, St. Louis, MO, USA). Helium 5.0 was purchased from Afrox (Cape Town, South Africa).

### 3.2. Method of cis/trans Ratio Determination in Standard Mixtures

The standards for linalool oxide (furanoid, *cis* and *trans*), pseudoionone (*cis* and *trans*) and farnesol (*E,Z; Z,E;* and *E,E*) contains a mixture of isomers. The standards were diluted to a concentration of 10,000 mg/L in deuterated chloroform. The ratio of *cis:trans* was identified using ^1^H nuclear magnetic resonance (NMR) spectroscopy. NMR spectra were recorded on a Varian unity Inova 300 (^1^H; 300.08 MHz) NMR spectrometer equipped with the sample injector at 30 °C.

Diastereomers containing the *cis/trans* double bond may be susceptible to interconversion under thermal treatment [20]. Terpenes in particular may be susceptible to this transformation, as demonstrated by multiple sources of literature [20,57,58,59]. Hence, to confirm the thermal stability of the *cis/trans* diastereomers at elevated temperatures (as the samples are introduced into the GC inlet at 250 °C), the ratio was also determined by GC–FID. The samples diluted to a concentration of 20 mg/L with dichloromethane and analysed by GC–FID. The ratio obtained from ^1^H NMR was compared to the ratio of *cis* and *trans* diastereomers identified by GC–FID. Importantly, no *cis:trans* inter-conversion was observed. The *cis:trans* ratio of linalool oxide (furanoid) and pseudoionone were 57:43 and 29:71 respectively. 

### 3.3. Method for Dearomatized Wines

White (Sauvignon Blanc) and red (Cabernet Sauvignon) wines were dearomatized by the following protocol: 500 mL of wine was reduced by rotary evaporation (58 mbar, water bath temperature 40 °C) for 30 min to yield a final volume of 400 mL. To this, 60 mL of absolute ethanol was added and the solution was made up to 500 mL with water (12% ethanol (*v*/*v*)).

### 3.4. Sample Preparation

#### 3.4.1. Offline SPE

The sample preparation was based on work by Piñeiro et al., with some modifications [19]. In summary, the SPE cartridge was conditioned with 4 mL dichloromethane and 4 mL methanol, followed by 4 mL model wine. To the 50 mL wine sample, 25 µL of 400 mg/L internal standard solution (3-octanol and 2,6-dimethyl-6-hepten-2-ol) was added. The wine sample was loaded onto the cartridge, washed with 6 mL of water which was followed by drying under vacuum (−60 KPa) for 25 min. The sample was eluted with 2 mL of DCM, dried over anhydrous sodium sulfate before analysis by GC–MS. 

#### 3.4.2. Online HS–SPME

The sample preparation and choice of fiber was based on literature, with some modifications [2,28,60]. In summary, to a 20 mL headspace vial, 2 g of NaCl was added. To this, 10 mL of wine was added, followed by the addition of 50 µL of the internal standard solution (100 mg/L 3-octanol and 2,6-dimethyl-6-hepten-2-ol in model wine). The samples were hermetically sealed followed by vortexing until the NaCl was dissolved before the online HS–SPME. The DVB/PDMS/CAR fiber (23 Ga.) was conditioned according to the manufacturer’s instructions before analysis. The sample was incubated for 5 min at 40 °C, followed by sample extraction with agitation (250 rpm; 3 s on time, 2 s off time) at 40 °C for 25 min, after which the sample was injected for GC–MS analysis. 

### 3.5. GC–MS Instrumental Parameters

GC–MS analysis was performed with a 7890B GC (Agilent, Palo Alto, CA, USA), equipped with a 5977B single quadrupole mass detector (Agilent, Palo Alto, CA, USA) and a PAL RSI 85 autosampler (CTC Analytics AG, Zwingen, Switzerland). Chromatographic separation was performed on a Zebron ZB-FFAP capillary column (60 m × 0.32 mm × 0.5 µm, Phenomenex, Torrence, CA, USA). The instrumental method was based on work by Marques and Jelen with the following changes [2,28]. The initial oven temperature was 40 °C held for 5 min, then ramped up to 120 °C at 8 °C/min, held for 4 min, then ramped to 170 °C at 5 °C/min and held for 4 minutes, followed by the final temperature ramp to 240 °C at 5 °C/min and held for 3 min. A post run at 240 °C at 4 mL/min for 5 min was included to thermally clean the column. Sample injection was done in the GC inlet port with the temperature maintained at 250 °C, conducted in splitless mode with the split flow set to 50 mL/min for 0.6 min. Gas saver was activated at 2 min at a flow rate of 20 mL/min. Helium was used as the carrier gas and the flow rate was set to 1.2 mL/min (constant flow). For liquid injections, a 1 µL volume in splitless mode was used; for HS–SPME injections, splitless mode was used with sample desorption time set at 10 min. The MS transfer line temperature was maintained at 250 °C.

Data was acquired in the single-ion monitoring (SIM) mode, with the solvent delay set at 12.5 minutes. MS source and quad temperatures were maintained at 230 and 150 °C, respectively, with the ionizing voltage set at 70 eV. For the instrumental method development stages, the analyses were performed in scan mode from 50 to 250 amu. The retention indices and spectra were compared to NIST11 spectral library. Details of the retention times and indices, quantifier and qualifier ions are listed in Table S3. Data analysis was performed with MassHunter qualitative (B.07.00) and quantitative (B.07.01) workstation software.

### 3.6. Method Performance Parameters 

The performance of the sample preparation methods evaluated the qualitative (selectivity) and quantitative (linearity, limits of detection (LOD), limits of quantitation (LOQ), precision and accuracy) parameters. 

#### 3.6.1. Selectivity

The selectivity was evaluated by spiking model wine and dearomatized white or red wine with the terpenoid stock solution to yield a final concentration of 2 and 20 µg/L for HS–SPME; 20 and 100 µg/L for SPE. The respective sample preparation and analysis was subsequently performed. The resultant chromatograms were compared, and the selectivity was evaluated by qualitatively comparing the results in the presence and absence of interferences from both red and white wine matrices. 

#### 3.6.2. Linearity

The linearity range was evaluated between 0.28 and 100 µg/L at seven concentrations in model, white and red wine for HS–SPME and 0.88 and 500 µg/L for SPE. The concentration range for the respective analytes are displayed in Table 2. Notably, for red and white wines, a blank deduction was performed to allow for comparison with model wine. The linearity correlation coefficients (R^2^) were calculated from regression analysis. 

#### 3.6.3. Limits of Detection and Limits of Quantitation

The LOD and LOQ were calculated as the lowest concentration of the analyte in a sample the resulted in a signal to noise ratio of 3 (LOD) and 10 (LOQ), respectively. The baseline noise was calculated by Agilent’s MassHunter software, the autoRMS algorithm was used to calculate the noise within the defined SIM time group for the selected quantitation ion (Table S3). For SPE extractions, the LOD and LOQ are reported in the original 50 mL sample.

#### 3.6.4. Accuracy (Recovery Test)

Accuracy was measured for two levels of the spiked concentration, namely 2 and 20 µg/L for HS–SPME and 20 and 100 µg/L for SPE—the results for which are displayed in Table S4 (Supplementary). Spiked model, red and white wines were extracted in triplicate.

#### 3.6.5. Precision (Repeatability Test)

Precision was determined by means as repeatability for the entire procedure, from sample preparation to instrument analysis. The repeatability (% relative standard deviation for each matrix) was measured in triplicate at 2 and 20 µg/L for HS–SPME and at 20 and 100 µg/L for SPE, and the results hereof are displayed in Table S5 (Supplementary). The RSD values were calculated for the response factors and retention times. 

## 4. Conclusions

The measurement of terpenoids was accomplished using both an offline SPE–GC–MS and online HS–SPME–GC–MS method. The sample preparation methods were applied to red, white and model wine, with good separation linearity, precision (repeatability) and accuracy (recovery). Notably, the online HS–SPME–GC–MS method proved to be more sensitive, faster and solvent-free, which is in line with green chemistry principles. To the best of our knowledge, the first *cis* and *trans* quantitation for pseudoionone was reported with concentrations ranging from nd to 1.2 µg/L using the HS–SPME method. The internal standards 3-octanol and 2,6-dimethyl-6-hepten-2-ol were compared, and both resulted in acceptable performance levels. Both sample preparation methods evaluated the terpenoid concentrations in white wines and the sum of the compounds ranged 13 to 232 µg/L for the offline SPE–GC–MS sample preparation method and from 15 to 192 µg/L for the online HS–SPME–GC–MS sample preparation method. 

## Figures and Tables

**Table 1:** Selected sensory parameters for terpenoids.

Compound	CAS Number	OTDs (µg/L)	Odor Descriptor
Limonene	5989-27-5	15 [39]	citrus [13]
6-Methyl-5-hepten-2-one	110-93-0	50 [40]	citrus-like [41]
Fenchone	7787-20-4	110 [42]	muddy [43], earthy [42]
*cis*-Linalool oxide (furanoid)	1365-19-1	-	elderflower, leaves, sweet [44]
*trans*-Linalool oxide (furanoid)	34995-77-2	320 [45]	woody [45]
Linalool	78-70-6	25 [46]	Floral [47], lavender [13]
Linalyl acetate	115-95-7	110 [48]	floral, sweet, mint, caraway-like [48]
α-terpineol	98-55-5	250 [46]	oily, anise, spicy [13]
β-Citronellol	106-22-9	18 [39]	citrus, floral [47]
Nerol	106-25-2	15 [39]	orange, floral, lemongrass [49]
β-Damascone	23726-91-2	-	fruity, floral, plum, rose [18]
β-Damascenone	23696-85-7	0.05 [50]	tobacco, apple, floral, rose [49]
Geraniol	106-24-1	30 [50]	floral [47], geranium [49]
α-Ionone	127-41-3	2.6 [46]	floral [49]
β-Ionone	14901-07-6	0.09 [46]	raspberry, floral [49]
*cis*-PseudoIonone	13927-47-4	800 [40]	-
*trans*-PseudoIonone	3548-78-5	800 [40]	-
Farnesol (*Z,E*)	3790-71-4	-	-
Farnesol (*E,Z*)	3879-60-5	-	-
Farnesol (*E,E*)	106-28-5	20 [45]	floral, oily, blueberry [51,52,53]

Odor thresholds are reported for a wine matrix except for 6-methyl-5-hepten-2-one (water), pseudoionone (water) and linalyl acetate (air).

**Table 2:** Selected method performance parameters for the quantitation of terpenoids in model (MW), red (RW) and white wine (WW) using both offline SPE and online HS–SPME GC–MS analysis.

Compound	Calibration Range (µg/L)			R^2^			Repeatability (RSD%) (*n* = 3)			Accuracy (%), Recovery Tests at 20 µg/L (*n* = 3)			LOD (µg/L)			LOQ (µg/L)		
HS–SPME																		
	**MW**	**WW**	**RW**	**MW**	**WW**	**RW**	**MW**	**WW**	**RW**	**MW**	**WW**	**RW**	**MW**	**WW**	**RW**	**MW**	**WW**	**RW**
**Limonene**	1–20	5–100	1–20	0.9806	0.9975	0.9946	1.9	8.1	6.4	112	108	110	0.0002	0.0019	0.0053	0.0008	0.0063	0.0178
**6-Methyl-5-hepten-2-one**	1–50	1–50	1–50	0.9967	0.9992	0.9988	1.5	2.4	1.8	105	98	104	0.0108	0.0209	0.2143	0.0361	0.0697	0.7144
**Fenchone**	1–50	1–50	1–50	0.9966	0.9987	0.9979	1.7	5.1	0.5	105	96	104	0.0036	0.0100	0.0208	0.0120	0.0334	0.0693
***cis*-Linalool oxide**	0.57–54	0.57–57	0.57–57	0.9991	0.9993	0.9996	1.8	1.2	2.3	104	88	102	0.0533	0.0687	0.1910	0.1777	0.2289	0.6366
***trans*-Linalool oxide**	0.43–43	0.43–43	0.43–43	0.9997	0.9995	0.9986	1.3	8.7	5.1	97	91	95	0.0298	0.0456	0.1804	0.0994	0.1521	0.6015
**Linalool**	1–50	1–50	1–50	0.9916	0.9961	0.9975	1.9	2.7	0.6	108	94	104	0.0154	0.0164	0.0584	0.0514	0.0545	0.1948
**Linalyl acetate**	5–100	5–100	10–100	0.9798	0.9913	0.9976	11.5	12.4	16.6	107	91	108	0.0157	0.0301	0.4197	0.0522	0.1003	1.3989
**α-Terpineol**	1–100	1–100	1–100	0.9974	0.9988	0.9997	1.2	2.8	0.7	104	87	100	0.0019	0.0076	0.0441	0.0064	0.0254	0.1470
**β-Citronellol**	1–100	1–100	1–100	0.9970	0.9996	0.9985	4.1	9.0	1.4	101	102	94	0.0218	0.0453	0.1477	0.0726	0.1512	0.4924
**Nerol**	1–100	1–100	1–100	0.9985	0.9991	0.9965	5.0	1.6	1.2	99	97	90	0.1049	0.2163	0.6350	0.3495	0.7210	2.1166
**β-Damascone**	1–100	1–100	1–100	0.9898	0.9983	0.9999	3.8	2.1	1.2	106	95	101	0.0005	0.0015	0.0043	0.0017	0.0051	0.0145
**β-Damascenone**	1–100	1–100	1–100	0.9919	0.9957	0.9994	3.9	1.3	1.2	105	98	103	0.0007	0.0019	0.0133	0.0024	0.0062	0.0442
**Geraniol**	1–100	1–100	1–100	0.9908	0.9978	0.9993	5.1	1.8	1.6	105	107	103	0.0972	1.1069	0.8291	0.3240	3.6898	2.7636
**α-Ionone**	1–100	1–100	1–100	0.9917	0.9988	0.9997	3.8	1.8	1.1	105	97	99	0.0007	0.0042	0.0067	0.0025	0.0141	0.0223
**β-Ionone**	1–100	1–100	1–100	0.9981	0.9998	0.9966	4.7	1.7	1.3	110	99	91	0.0006	0.0020	0.0069	0.0020	0.0066	0.0231
***cis*-Pseudoionone**	0.28–27.6	0.28–13.82	0.28–13.82	0.9931	0.9919	0.9876	5.4	1.1	3.1	89	100	90	0.0073	0.0349	0.1786	0.0244	0.1162	0.5954
***trans*-Pseudoionone**	0.73–72.8	0.73–36.4	0.73–36.4	0.9957	0.9977	0.9909	7.0	0.7	4.6	93	103	92	0.0101	0.0465	0.2582	0.0338	0.1550	0.8606
**Farnesol (*Z,E*)**	0.088–8.79	0.18–4.4	0.18–4.4	0.9962	0.9907	0.9434	3.3	4.6	6.2	98	106	87	0.0631	0.0322	0.1842	0.2104	0.1073	0.6141
**Farnesol (*E,Z*)**	0.35–35	0.70–17.5	0.35–17.5	0.9952	0.9967	0.9830	3.2	2.5	8.0	98	107	87	0.0387	0.0473	0.2436	0.1290	0.1575	0.8118
**Farnesol (*E,E*)**	0.57–56.7	0.57–56.7	0.57–28.34	0.9951	0.9958	0.9907	2.6	3.4	5.8	98	109	82	0.0207	0.0419	0.2773	0.0692	0.1395	0.9245
**SPE**																		
	MW	WW	RW	MW	WW	RW	MW	WW	RW	MW	WW	RW	MW	WW	RW	MW	WW	RW
**Limonene**	5–100	5–100	5–100	0.9997	0.9934	0.9853	8.4	7.9	8.3	104	119	119	0.0226	0.0399	0.0504	0.0755	0.1330	0.1680
**6-Methyl-5-hepten-2-one**	5–500	5–500	5–500	0.9993	0.9993	0.9987	7.0	3.8	5.9	101	98	102	0.0098	0.0137	0.0135	0.0328	0.0457	0.0450
**Fenchone**	5–500	5–500	5–500	0.9994	0.9991	0.9989	6.4	4.6	5.4	98	98	101	0.0123	0.0139	0.0146	0.0412	0.0464	0.0488
***cis*-Linalool oxide**	2.87–287	2.87–287	2.87–287	0.9997	0.998	0.9993	3.8	4.0	6.1	100	93	90	0.0349	0.0640	0.0587	0.1162	0.2133	0.1958
***trans*-Linalool oxide**	2.15–215	2.15–215	2.15–215	0.9958	0.9979	0.9993	3.7	3.7	5.2	101	93	90	0.0361	0.0679	0.0627	0.1203	0.2265	0.2091
**Linalool**	5–500	5–500	5–500	0.9988	0.9987	0.9983	4.3	4.1	3.6	96	94	96	0.0662	0.0279	0.0006	0.2205	0.0931	0.0021
**Linalyl acetate**	5–500	5–500	5–500	0.9959	0.9978	0.9992	4.7	7.0	3.4	90	92	100	0.0445	0.0323	0.0369	0.1483	0.1077	0.1229
**α-Terpineol**	5–500	5–500	5–500	0.9987	0.9983	0.9996	5.3	5.5	3.4	98	96	101	0.0346	0.1404	0.0151	0.1152	0.4679	0.0504
**β-Citronellol**	5–500	5–500	5–500	0.9987	0.9980	0.9987	6.5	3.6	7.4	90	91	102	0.1458	0.1078	0.0880	0.4859	0.3595	0.2935
**Nerol**	5–500	5–500	5–500	0.9995	0.9985	0.9995	3.7	4.1	4.6	91	91	96	0.2273	0.1985	0.2093	0.7575	0.6618	0.6977
**β-Damascone**	5–500	5–500	5–500	0.9994	0.9988	0.9995	6.9	4.2	3.5	95	94	102	0.0074	0.0074	0.0073	0.0247	0.0248	0.0242
**β-Damascenone**	5–500	5–500	5–500	0.9993	0.9987	0.9994	6.5	4.2	3.1	95	94	103	0.0087	0.0088	0.0095	0.0289	0.0294	0.0317
**Geraniol**	5–500	5–500	5–500	0.9989	0.9987	0.9998	6.0	5.6	5.7	91	94	100	0.3121	0.2234	0.2554	1.0402	0.7447	0.8515
**α-Ionone**	5–500	5–500	5–500	0.9994	0.9985	0.9996	6.8	4.0	3.6	93	92	100	0.0097	0.0105	0.0122	0.0323	0.0351	0.0407
**β-Ionone**	5–500	5–500	5–500	0.9996	0.9987	0.9995	6.6	4.1	4.8	95	93	103	0.0057	0.0059	0.0061	0.0188	0.0196	0.0202
***cis*-Pseudoionone**	1.38–138	1.38–138	1.38–138	0.998	0.9961	0.9983	6.2	7.6	5.1	88	88	97	0.0461	0.0826	0.0714	0.1538	0.2752	0.2381
***trans*-Pseudoionone**	3.63–363	3.63–363	3.63–363	0.9992	0.9981	0.9996	6.3	4.2	5.7	90	89	101	0.0343	0.0594	0.0564	0.1143	0.1980	0.1879
**Farnesol (*Z,E*)**	0.88–441	0.88–441	0.88–441	0.9978	0.9991	0.9990	3.1	7.6	7.9	94	91	96	0.1079	0.1553	0.0665	0.3596	0.5177	0.2218
**Farnesol (*E,Z*)**	1.75–175	1.75–175	1.75–175	0.9971	0.9965	0.9990	7.9	1.0	6.5	85	81	98	0.1320	0.1865	0.0792	0.4400	0.6218	0.2642
**Farnesol (*E,E*)**	2.8–280	2.8–280	2.8–280	0.9954	0.9974	0.9978	7.0	5.2	6.0	83	83	101	0.1202	0.1464	0.0513	0.4006	0.4881	0.1709

**Table 3:** Description and terpenoid quantitation (expressed in µg/L) in white wine^1^ using the online HS–SPME sample preparation method and offline SPE sample preparation method, respectively.

Online HS–SPME														
	FLY16S	ARV16S	ANI16/17S	JCB16CH	FRN17SB	FLK17AL	KZC18CB	KLA16S	ORB17AL	LNC17SB	ARV16S/B	GWZ19C	GWC19E	MRF17SB
**Limonene**	<LOQ	<LOQ	<LOQ	<LOQ	<LOQ	0.27	<LOQ	<LOQ	<LOQ	<LOQ	24.39	0.13	0.01	<LOQ
**6-Methyl-5-hepten-2-one**	<LOQ	<LOQ	0.06	<LOQ	<LOQ	0.8	0.46	<LOQ	<LOQ	<LOQ	0.31	0.87	1.06	<LOQ
**Fenchone**	<LOQ	<LOD	<LOD	<LOQ	<LOQ	<LOQ	<LOQ	<LOQ	<LOQ	<LOQ	<LOQ	<LOQ	<LOQ	<LOQ
***cis*-Linalool oxide**	20.79	8.1	18.93	6.58	4.84	20.56	<LOD	19.08	2.84	10.33	14.01	5.53	2.34	3.69
***trans*-Linalool oxide**	6.14	3.07	5.96	2.84	1.77	21.38	0.78	5.38	4.71	3.86	4.81	3.24	2.22	1.7
**Linalool**	0.29	0.82	1.31	1.93	2.06	37.9	2.44	2.13	15.22	3.49	0.1	22.04	14.6	3.96
**Linalyl acetate**	<LOQ	<LOQ	<LOQ	<LOD	<LOD	<LOD	<LOD	<LOQ	<LOD	<LOQ	<LOQ	<LOQ	<LOQ	<LOD
**α-Terpineol**	2.92	4.03	4.11	4.97	3.18	56.23	0.91	4.73	11.79	12.68	5.21	10.76	6.3	3.46
**β-Citronellol**	1.35	0.76	1.73	1.81	1.75	5.27	2.13	1.61	3.13	2.24	0.52	34	75.43	3.69
**Nerol**	0.76	2.08	<LOD	5.05	5.55	7.82	0.76	3.46	2.92	7.19	2.1	13.78	21.04	<LOD
**β-Damascone**	0.01	<LOQ	<LOQ	<LOQ	<LOQ	<LOQ	<LOQ	<LOQ	<LOQ	<LOQ	<LOQ	<LOQ	<LOQ	<LOQ
**β-Damascenone**	0.64	0.75	0.66	0.74	0.27	1.31	0.35	1.69	1.68	<LOQ	2.45	3.24	1.75	1.45
**Geraniol**	3.27	64.39	9.45	5.41	6.49	20.21	3.48	3.56	8.67	7.77	78.95	60.22	63.37	13.14
**α-Ionone**	0.04	<LOQ	<LOQ	<LOQ	<LOQ	<LOQ	<LOQ	<LOQ	<LOQ	<LOQ	<LOQ	<LOQ	<LOQ	<LOQ
**β-Ionone**	0.26	0.06	0.04	0.13	0.03	0.03	0.03	0.04	0.03	0.01	0.32	0.02	0.02	0.03
***cis*-Pseudoionone**	<LOD	<LOD	<LOD	<LOD	<LOD	<LOD	<LOD	<LOD	<LOD	<LOD	0.36	<LOD	<LOD	<LOD
***trans*-Pseudoionone**	0.27	0.34	1.02	0.35	0.23	0.18	0.32	0.2	0.16	0.19	1.11	0.29	0.29	0.28
**Farnesol (*Z,E*)**	0.11	<LOD	0.05	0.63	0.1	0.29	0.21	0.06	0.42	0.03	0.03	0.01	0.01	0.62
**Farnesol (*E,Z*)**	<LOD	<LOD	<LOD	<LOD	<LOD	<LOD	<LOD	<LOD	<LOD	<LOD	0.32	<LOD	<LOD	<LOD
**Farnesol (*E,E*)**	0.15	0.11	0.4	1.77	0.53	0.69	3.66	0.09	1.87	0.06	0.91	3.12	3.24	1.87
**Offline SPE**														
	**FLY16S**	**ARV16S**	**ANI16/17S**	**JCB16CH**	**FRN17SB**	**FLK17AL**	**KZC18CB**	**KLA16S**	**ORB17AL**	**LNC17SB**	**ARV16S/B**	**GWZ19C**	**GWC19E**	**MRF17SB**
**Limonene**	<LOQ	<LOQ	<LOQ	<LOQ	<LOQ	<LOQ	<LOQ	<LOQ	<LOQ	<LOQ	<LOQ	<LOQ	<LOQ	<LOQ
**6-Methyl-5-hepten-2-one**	<LOD	<LOD	<LOQ	0.02	<LOD	0.91	0.53	<LOD	<LOD	<LOD	0.07	1.68	1.63	<LOD
**Fenchone**	<LOD	<LOD	<LOD	<LOD	<LOD	<LOD	<LOD	<LOD	<LOD	<LOD	<LOD	<LOD	<LOD	<LOD
***cis*-Linalool oxide**	24.5	11.01	19.81	11.12	8.11	30.41	0.07	16.48	4.24	18.26	12.89	1.39	0.87	5.17
***trans*-Linalool oxide**	8.23	3.48	5.84	4.1	2.7	21.62	0.19	5.15	1.32	4.65	4.33	1.45	1.18	1.19
**Linalool**	1.45	1.66	2.37	4.22	3.41	63.16	4.04	8.02	21.51	5.33	0.62	19.09	12.43	6.01
**Linalyl acetate**	<LOD	<LOD	<LOD	<LOD	<LOD	<LOD	<LOD	<LOD	<LOD	<LOD	<LOD	<LOD	<LOD	<LOD
**α-Terpineol**	3.46	4.7	4.87	5.97	2.99	85.92	<LOQ	7.01	14.83	18.97	6.02	3.81	1.72	3.4
**β-Citronellol**	2.12	1.47	2.36	2.35	2.32	5.98	2.64	2.81	3.29	2.68	1.23	48.86	92.34	3.7
**Nerol**	<LOD	<LOD	<LOD	<LOD	<LOD	5.92	<LOD	<LOD	2.88	<LOD	<LOD	18	24.25	<LOD
**β-Damascone**	<LOQ	<LOQ	<LOQ	<LOQ	<LOQ	<LOQ	<LOQ	<LOQ	<LOQ	<LOQ	<LOQ	<LOQ	<LOQ	<LOQ
**β-Damascenone**	0.98	1.19	1.19	1.07	0.71	1.61	0.86	2.42	1.68	0.36	2.97	3.79	2.01	1.47
**Geraniol**	<LOD	9.63	0.42	1.56	1.71	14.72	2.83	<LOD	6.01	3.5	13.68	66.92	60.07	4.78
**α-Ionone**	<LOD	<LOD	<LOD	<LOD	<LOD	<LOD	<LOD	<LOD	<LOD	<LOD	<LOD	0.79	0.75	<LOD
**β-Ionone**	<LOD	<LOD	<LOD	<LOD	<LOD	0.42	<LOD	0.47	<LOD	<LOD	0.51	<LOD	<LOD	<LOD
***cis*-Pseudoionone**	<LOD	<LOD	<LOD	<LOD	<LOD	<LOD	<LOD	<LOD	<LOD	<LOD	<LOD	1.48	1.41	<LOD
***trans*-Pseudoionone**	0.72	<LOD	<LOD	<LOD	<LOD	<LOD	<LOD	<LOD	<LOD	<LOD	<LOD	0.74	<LOD	<LOD
**Farnesol (*Z,E*)**	0.59	<LOD	<LOD	0.62	<LOD	0.58	<LOD	<LOD	0.73	0.7	<LOD	0.68	0.71	0.85
**Farnesol (*E,Z*)**	<LOD	<LOD	<LOD	<LOD	<LOD	<LOD	<LOD	<LOD	<LOD	<LOD	<LOD	0.77	<LOD	<LOD
**Farnesol (*E,E*)**	0.13	0.23	0.24	0.91	0.51	0.59	2.44	0.42	1.36	0.19	0.16	12.06	12.07	1.84

^1^ See Table S2 in Supplementary Materials section for the wine description.

## Supplementary Materials

The following are available online at https://www.mdpi.com/1420-3049/25/3/657/s1, **Figure S1a**) SPE GC-MS chromatogram in spiked model wine (20 µg/L), spiked dearomatised white (20 µg/L) and a real white wine sample (ARV16S/B) respectively. **Figure S1b**) HS-SPME GC-MS chromatogram in spiked model wine (20 µg/L), spiked dearomatised white (20 µg/L) and a real white wine sample (ARV16S/B) respectively. **Table S1.** Precision and accuracy results for the comparison of 3-octanol and 2,6-dimethyl-6-hepten-2-ol as internal standards. **Table S2.** Description and alcohol concentration of selected white wines used in this study. **Table S3.** Calculated and literature retention indices for the analyses of terpenoids using both HS-SPME and SPE. **Table S4.** Accuracy results by means of recovery (%) for terpenoids quantitation at 100 µg/L for SPE and 2 µg/L for SPME for model (MW), white (WW) and red (RW) wine respectively. **Table S5.** Precision results by means of repeatability (%) for terpenoids quantitation at 100 µg/L for SPE and 2 µg/L for SPME for model (MW), white (WW) and red (RW) wine respectively.
