# Supplementary material for: Comparison of an Offline SPE–GC–MS and Online HS–SPME–GC–MS Method for the Analysis of Volatile Terpenoids in Wine
Source: Molecules. 2020 Feb 4;25(3):657. doi: 10.3390/molecules25030657 (PMC7036967; doi:10.3390/molecules25030657)
Supplement: Supplementary file 1 [file molecules-25-00657-s001.pdf]

*molecules*

MDPI

*Article*

# Comparison of an offline-SPE-GC-MS and online-HS-SPME-GC-MS method for the analysis of volatile terpenoids in wine

**Cody Williams[1] and Astrid Buica[1,*]**

[1] Department of Viticulture and Oenology, Stellenbosch University, 7600 Stellenbosch, RSA
* Author to whom correspondence should be addressed: abuica@sun.ac.za

**Supplementary Materials:** The following are available online at www.mdpi.com/xxx/s1.

**Figure S1a and S1b)** The offline-SPE GC-MS chromatogram in spiked model wine (20 µg/L), spiked dearomatised white (20 µg/L) and a real white wine sample (ARV16S/B) respectively. **Figure S1b)** The online-HS-SPME GC-MS chromatogram in spiked model wine (20 µg/L), spiked dearomatised white (20 µg/L) and a real white wine sample (ARV16S/B) respectively.

**Table S1.** Precision and recovery (accuracy) results for the comparison of 3-octanol and 2,6-dimethyl-6-hepten-2-ol as internal standards.

**Table S2.** Description and alcohol concentration of selected white wines used in this study.

**Table S3.** Calculated and literature retention indices for the analyses of terpenoids using both HS-SPME and SPE.

**Table S4.** Accuracy results by means of recovery (%) for terpenoids quantitation at 100 µg/L for SPE and 2 µg/L for SPME for model (MW), white (WW) and red (RW) wine respectively.

**Table S5.** Precision results by means of repeatability (%) for terpenoids quantitation at 100 µg/L for SPE and 2 µg/L for SPME for model (MW), white (WW) and red (RW) wine respectively.

molecules

MDPI

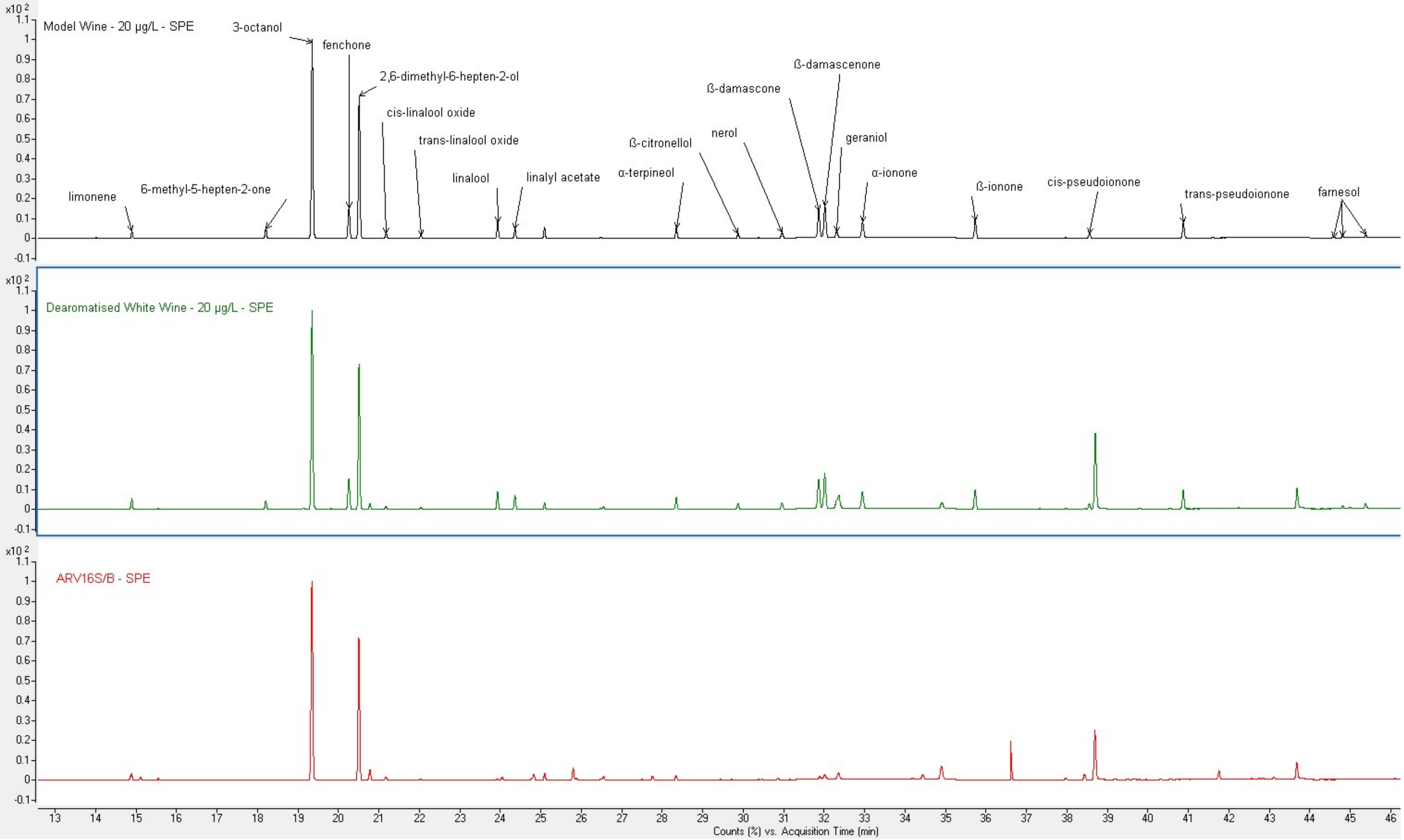


**Figure S1a)** The offline-SPE GC-MS chromatogram in spiked model wine (20 µg/L), spiked dearomatised white (20 µg/L) and a real white wine sample (ARV16S/B) respectively.

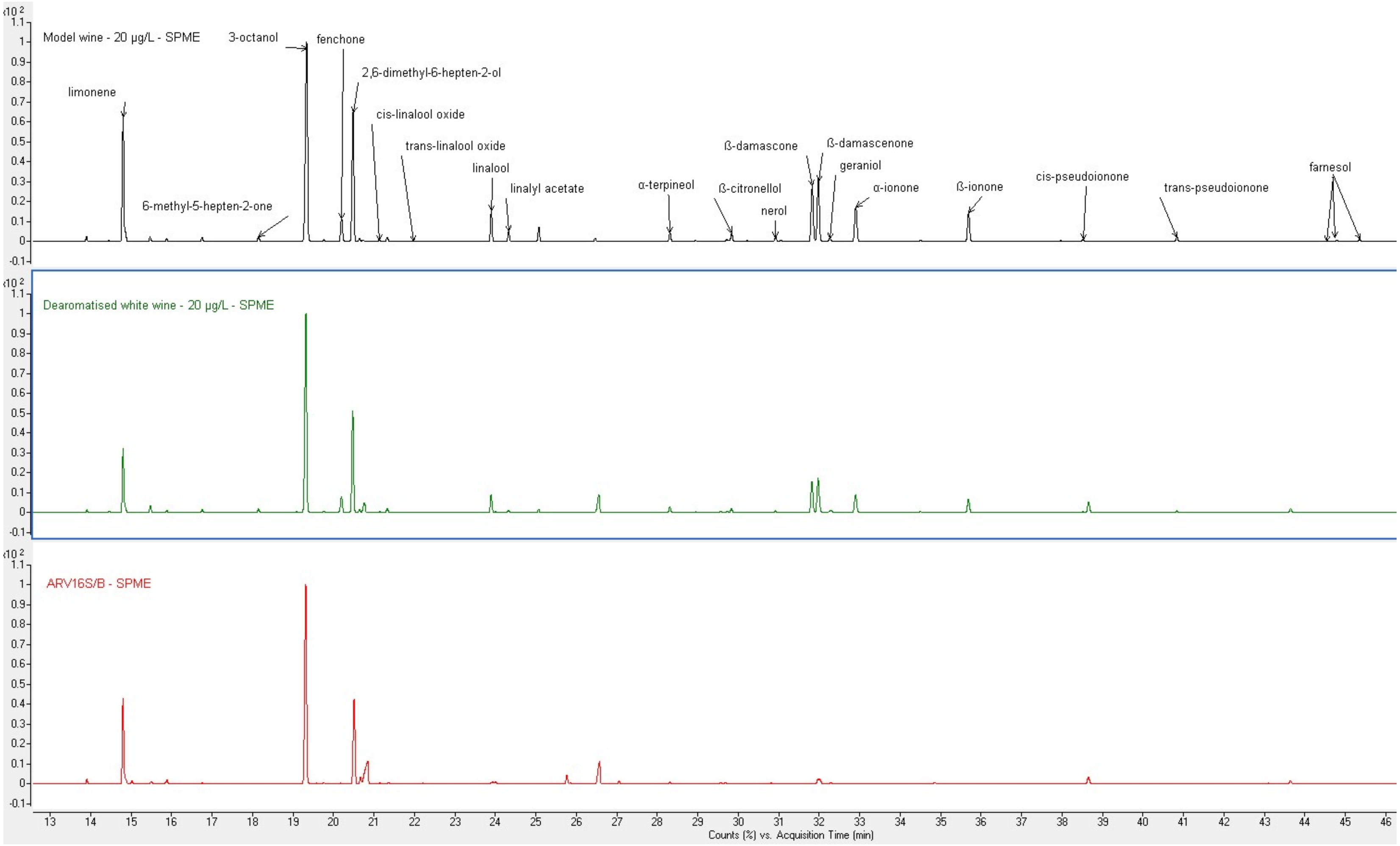


**Figure S1b.** The online-HS-SPME GC-MS chromatogram in spiked model wine (20 µg/L), spiked dearomatised white (20 µg/L) and a real white wine sample (ARV16S/B) respectively.

**Table S1.** Precision and recovery (accuracy) results for the comparison of 3-octanol and 2,6-dimethyl-6-hepten-2-ol as internal standards.

| | | SPE | | | SPME | | |
|---|---|---|---|---|---|---|---|
| | | **MW** | **WW** | **RW** | **MW** | **WW** | **RW** |
| 3-Octanol (IS-1) | Precision[a] (%) | 10.99 | 7.36 | 9.93 | 8.56 | 8.51 | 5.08 |
| | Rt (%) | 0.00591 | 0.00554 | 0.00635 | 0.00851 | 0.0106 | 0.00687 |
| | $R^2$ (min) | 0.9954 | 0.9934 | 0.9853 | 0.9798 | 0.9907 | 0.9434 |
| | $R^2$ (max) | 0.9997 | 0.9993 | 0.9998 | 0.9997 | 0.9998 | 0.9999 |
| | Recovery - min (%) | 83.20 | 81.03 | 89.81 | 88.96 | 87.32 | 82.37 |
| | Recovery - max (%) | 103.69 | 118.7 | 118.9 | 112.17 | 108.84 | 110.34 |
| 2,6-dimethyl-6-hepten-2-ol (IS-2) | Precision[a] (%) | 12.63 | 7.96 | 11.83 | 12.89 | 9.908 | 6.054 |
| | Retention time RSD (%) | 0.00724 | 0.00469 | 0.00717 | 0.00685 | 0.0125 | 0.00737 |
| | $R^2$ (min) | 0.9912 | 0.9812 | 0.9839 | 0.9506 | 0.9814 | 0.9638 |
| | $R^2$ (max) | 0.9994 | 0.9997 | 0.9988 | 0.9983 | 0.9997 | 0.9996 |
| | Recovery - min (%) | 82.53 | 20.17 | 91.43 | 88.09 | 86.48 | 89.31 |
| | Recovery - max (%) | 101.91 | 134.67 | 136.5 | 149.89 | 148.25 | 149.31 |

[a] – Refers to the combined sample preparation and instrumental repeatability RSD (%)

[b] – Rt refers to the retention time RSD (%)

**Table S2.** Description and alcohol concentration of selected white wines used in this study.

| Sample Code | Cultivar | Vintage | Alcohol (%) |
|---|---|---|---|
| FLY16S | Solaris | 2016 | 14.5 |
| ARV16S | Solaris | 2016 | 14.0 |
| ANI16/17S | Solaris | 2016/2017 | 14.5 |
| JCB16CH | Chardonnay | 2016 | 12.5 |
| FRN17SB | Sauvignon Blanc | 2017 | 12.5 |
| FLK17AL | Albariño | 2017 | 13.0 |
| KZC18CB | Chenin Blanc | 2018 | 13.0 |
| KLA16S | Solaris | 2016 | 16.0 |
| ORB17AL | Albariño | 2017 | 12.5 |
| LNC17SB | Sauvignon Blanc | 2017 | 11.5 |
| ARV16S/B | Solaris Barrique | 2016 | 14.0 |
| GWZ19C | Gewürztraminer | 2019 | 11.0 |
| GWC19E | Gewürztraminer | 2019 | 11.0 |
| MRF17SB | Sauvignon Blanc | 2017 | 13.5 |

**Table S3.** Calculated (cRI) and literature retention indices (RI) for the analyses of terpenoids using both online HS-SPME and offline-SPE sample preparation methods.

| Compound | RT (min) | cRI | RI | Ions (m/z) | |
|---|---|---|---|---|---|
| | | (calc.) | (lit.) | Quant | Qual |
| (R)-(+)-Limonene | 14.891 | 1220 | 1219 | 93 | 68 |
| 6-Methyl-5-hepten-2-one | 18.207 | 1361 | 1359 | 108 | 111 |
| Fenchone | 20.260 | 1435 | 1422 | 152 | 81 |
| *cis*-Linalool oxide (furanoid) | 21.178 | 1466 | 1467 | 94 | 111 |
| *trans*-Linalool oxide (furanoid) | 22.036 | 1495 | 1490 | 94 | 111 |
| Linalool | 23.933 | 1562 | 1561 | 71 | 121 |
| Linalyl acetate | 24.364 | 1577 | 1575 | 121 | 93 |
| $\alpha$-terpineol | 28.347 | 1724 | 1724 | 136 | 59 |
| β-Citronellol | 29.872 | 1783 | 1782 | 123 | 95 |
| Nerol | 30.966 | 1821 | 1806 | 123 | 95 |
| β-Damascone | 31.864 | 1851 | 1824 | 177 | 192 |
| β-Damascenone | 32.018 | 1856 | 1827 | 121 | 190 |
| Geraniol | 32.312 | 1866 | 1832 | 121 | 84 |
| $\alpha$-Ionone | 32.948 | 1887 | 1844 | 121 | 192 |
| β-Ionone | 35.731 | 1879 | 1889 | 177 | 192 |
| *cis*-PseudoIonone | 38.556 | - | - | 69 | 124 |
| *trans*-PseudoIonone | 40.872 | - | 2073 | 69 | 124 |
| Farnesol (*Z*,*E*) | 44.578 | - | 2355 | 69 | 81 |
| Farnesol (*E*,*Z*) | 44.816 | - | 2329 | 69 | 81 |
| Farnesol (*E*,*E*) | 45.382 | - | 2355 | 69 | 81 |

**Table S4.** Accuracy results by means of recovery (%) for terpenoids quantitation at 100 μg/L for SPE and 2 μg/L for SPME for model (MW), white (WW) and red (RW) wine respectively.

| Accuracy (recovery, n = 3) | SPE | | SPME | |
|---|---|---|---|---|
| | 100 μg/L | | 2 μg/L | |
| | WW | RW | WW | RW |
| Limonene | 82 | 122 | 107 | 114 |
| 6-Methyl-5-hepten-2-one | 102 | 109 | 133 | 145 |
| Fenchone | 95 | 94 | 124 | 141 |
| cis-Linalool oxide | 152 | 147 | 84 | 95 |
| trans-Linalool oxide | 161 | 155 | 98 | 94 |
| Linalool | 105 | 92 | 118 | 131 |
| Linalyl acetate | 75 | 81 | 142 | 123 |
| α-Terpineol | 110 | 99 | 108 | 124 |
| β-Citronellol | 106 | 94 | 105 | 152 |
| Nerol | 104 | 91 | 104 | 132 |
| β-Damascone | 95 | 90 | 102 | 128 |
| β-Damascenone | 93 | 90 | 92 | 106 |
| Geraniol | 96 | 86 | 101 | 146 |
| α-Ionone | 95 | 92 | 106 | 141 |
| β-Ionone | 94 | 89 | 116 | 116 |
| cis-Pseudoionone | 104 | 93 | 159 | 145 |
| trans-Pseudoionone | 98 | 91 | 120 | 111 |
| Farnesol (*Z*,*E*) | 83 | 77 | 139 | 128 |
| Farnesol (*E*,*Z*) | 85 | 78 | 108 | 130 |
| Farnesol (*E*,*E*) | 80 | 73 | 120 | 107 |

**Table S5.** Precision results by means of repeatability (%) for terpenoids quantitation at 100 μg/L for SPE and 2 μg/L for SPME for model (MW), white (WW) and red (RW) wine respectively.

| Precision (repeatability, n = 3) | SPE | | | SPME | | |
|---|---|---|---|---|---|---|
| | 100 μg/L | | | 2 μg/L | | |
| | MW | WW | RW | MW | WW | RW |
| Limonene | 11.9 | 8.3 | 6.8 | 5.9 | 5.7 | 5.1 |
| 6-Methyl-5-hepten-2-one | 3.9 | 6.6 | 5.9 | 0.9 | 3.3 | 2.3 |
| Fenchone | 3.9 | 6.0 | 5.7 | 1.6 | 3.8 | 8.7 |
| cis-Linalool oxide | 2.9 | 6.6 | 3.6 | 2.8 | 1.6 | 4.1 |
| trans-Linalool oxide | 2.9 | 6.5 | 3.9 | 3.0 | 1.6 | 4.4 |
| Linalool | 3.5 | 4.9 | 4.9 | 2.4 | 4.1 | 3.9 |
| Linalyl acetate | 3.4 | 6.2 | 6.5 | 9.6 | 13.3 | 9.8 |
| α-Terpineol | 2.9 | 4.6 | 5.3 | 6.4 | 0.8 | 3.6 |
| β-Citronellol | 3.2 | 4.7 | 4.8 | 4.9 | 4.6 | 6.4 |
| Nerol | 3.0 | 4.6 | 6.3 | 5.5 | 5.6 | 8.5 |
| β-Damascone | 3.6 | 5.4 | 6.7 | 4.1 | 1.2 | 7.4 |
| β-Damascenone | 3.8 | 5.6 | 6.7 | 3.5 | 1.1 | 8.1 |
| Geraniol | 2.7 | 5.5 | 6.2 | 4.4 | 8.9 | 16.1 |
| α-Ionone | 3.8 | 5.4 | 6.7 | 5.1 | 1.2 | 8.1 |
| β-Ionone | 3.5 | 5.3 | 6.9 | 6.7 | 1.5 | 8.2 |
| cis-Pseudoionone | 3.7 | 5.4 | 7.9 | 9.0 | 7.0 | 6.0 |
| trans-Pseudoionone | 3.3 | 5.7 | 8.0 | 10.0 | 14.6 | 12.7 |
| Farnesol (*Z*,*E*) | 4.5 | 5.5 | 8.3 | 18.2 | 7.2 | 27.6 |
| Farnesol (*E*,*Z*) | 3.0 | 6.0 | 8.4 | 17.0 | 16.7 | 15.6 |
| Farnesol (*E*,*E*) | 3.0 | 6.5 | 10.7 | 18.0 | 15.8 | 14.1 |

© 2019 by the authors. Submitted for possible open access publication under the terms and conditions of the Creative Commons Attribution (CC BY) license (http://creativecommons.org/licenses/by/4.0/).
